# It is buzziness time: rearing, mating, and overwintering *Bombus vosnesenskii* (Hymenoptera: Apidae)

**DOI:** 10.1093/jisesa/iead089

**Published:** 2023-10-07

**Authors:** Morgan E Christman, N Pinar Barkan, Claire Campion, Sam D Heraghty, Ellen C Keaveny, Kelton M Verble, Sarah A Waybright, Michael E Dillon, Jeffrey D Lozier, James P Strange

**Affiliations:** Department of Entomology, The Ohio State University, Columbus, OH 43210, USA; Department of Entomology, The Ohio State University, Columbus, OH 43210, USA; Department of Zoology and Physiology, Program in Ecology, University of Wyoming, Laramie, WY 82071, USA; Department of Biological Sciences, University of Alabama, Tuscaloosa, AL 35487, USA; Department of Zoology and Physiology, Program in Ecology, University of Wyoming, Laramie, WY 82071, USA; Department of Biological Sciences, University of Alabama, Tuscaloosa, AL 35487, USA; Department of Zoology and Physiology, Program in Ecology, University of Wyoming, Laramie, WY 82071, USA; Department of Zoology and Physiology, Program in Ecology, University of Wyoming, Laramie, WY 82071, USA; Department of Biological Sciences, University of Alabama, Tuscaloosa, AL 35487, USA; Department of Entomology, The Ohio State University, Columbus, OH 43210, USA

**Keywords:** bumble bee, nest initiation, nest establishment, cold storage, CO_2_ narcosis

## Abstract

*Bombus vosnesenskii* Radowszkowski, 1862 is one of three bumble bee species commercially available for pollination services in North America; however, little is documented about *B. vosnesenskii* colony life cycle or the establishment of ex situ rearing, mating, and overwintering practices. In this study, we documented nest success, colony size, and gyne production; recorded the duration of mating events; assessed overwintering survival of mated gynes; and evaluated second-generation nest success for colonies established from low- and high-elevation wild-caught *B. vosnesenskii* gynes. Of the 125 gynes installed, 62.4% produced brood cells (nest initiation) and 43.2% had at least 1 worker eclose (nest establishment). High-elevation *B. vosnesenskii* gynes had significantly higher nest initiation and establishment success than low-elevation gynes. However, low-elevation colonies were significantly larger with queens producing more gynes on average. Mating was recorded for 200 low-elevation and 37 high-elevation gynes, resulting in a mean duration of 62 and 51 min, respectively. Mated gynes were then placed into cold storage for 54 days to simulate overwintering, which resulted in 59.1% of low-elevation gynes surviving and 91.9% of high-elevation gynes surviving. For second-generation low-elevation gynes, 26.4% initiated nesting and 14.3% established nesting. Second-generation high-elevation gynes did not initiate nesting despite CO_2_ narcosis treatments. Overall, these results increase our understanding of *B. vosnesenskii* nesting, mating, and overwintering biology from 2 elevations. Furthermore, this study provides information on successful husbandry practices that can be used by researchers and conservationists to address knowledge gaps and enhance the captive rearing of bumble bees.

## Introduction

The yellow-faced bumble bee (*Bombus vosnesenskii* Radowszkowski, 1862) is commonly found in open grassy areas, urban parks and gardens, shrubland, and mountains across low- and high-elevation areas throughout California, Oregon, and Washington, USA and southern British Columbia, Canada (Williams et al. 2014). *Bombus vosnesenskii* commonly forage on species of *Acrtostaphylos*, *Ceanothus*, *Chrysothamnus*, *Cirsium*, *Clarkia*, *Ericameria*, *Eriogonum*, *Eschscholzia*, *Grindelia*, *Lupinus*, *Phacelia*, *Rhododendron*, *Ribes*, and *Vicia* (Koch et al. 2012, Williams et al. 2014). Additionally, as a buzz pollinator, *B. vosnesenskii* was established as a successful greenhouse pollinator of tomato crops (Dogterom et al. 1998, Strange 2015), making it a viable candidate for commercialization.

*Bombus vosnesenskii* is now one of 2 bumble bee species currently available for commercial production in the United States, where it can be purchased for crop pollination throughout its native range (Koppert 2022). Although, *B. vosnesenskii* are commercially produced, little information is publicly available regarding rearing methods, nest success, and developmental timelines (Rowe et al. 2023, Strange et al. 2023). Previous research identified that *B. vosnesenskii* have high nest success in a controlled laboratory setting (Rowe et al. 2023, Strange et al. 2023). For example, from 2014 to 2019, 48.2% of wild-caught *B. vosnesenskii* gynes produced brood (nest initiation), and 25.2% had one worker eclose (nest establishment) (Strange et al. 2023). These *B. vosnesenskii* queens produced large colonies with an average of 238.8 emerged worker/male brood cells and 5.7 emerged gyne cells per colony (Strange et al. 2023). To date, no information has been published on mating or overwintering methodology or success, likely due to the proprietary nature of industry-based practices. Furthermore, differences in success and size between colonies reared from gynes collected from low- and high-elevation sites have not been evaluated.

As elevational generalists, *B. vosnesenskii* are adept at inhabiting environmentally and spatially heterogeneous landscapes (Lozier et al. 2021). Previous studies have identified that evaluating species across elevational gradients provides a unique opportunity to determine intraspecific differences in morphological traits, population dynamics, gene flow, defense mechanisms, and physiology (Dillon et al. 2006, Oyen et al. 2016, Ramírez-Delgado et al. 2016, Gérard et al. 2018, Dillon and Lozier 2019, Barkan et al. 2020, Pimsler et al. 2020, Lozier et al. 2021). Therefore, it is likely that colony development and structure would also vary along this gradient to account for differences in environmental factors (e.g., weather/climate, seasonality), but additional research is needed.

With the recent development of *B. vosnesenskii* as a commercial pollinator and interest in this species for scientific research (Pimsler et al. 2020, Lozier et al. 2021, Oyen et al. 2021), the need for public data on ex situ rearing, mating, and overwintering methods and success has become critical. In this study, we evaluated nest initiation and establishment success and documented colony size and gyne production to create a timeline of colony development for *B. vosnesenskii* colonies produced from low- and high-elevation wild-caught gynes. Additionally, we established successful captive mating techniques and documented the duration of mating events for newly eclosed low- and high-elevation gynes. We also evaluated the survival rates of low- and high-elevation mated gynes within 2 cold storage treatments. Furthermore, we assessed second-generation nest success for colonies established from low- and high-elevation wild-caught gynes and examined the impact of CO_2_ narcosis on nest initiation. These results contribute to increased knowledge of the systematic nesting, mating, and overwintering biology of *B. vosnesenskii* from low- and high-elevation sites in Oregon, USA, under captive rearing conditions as well as enhance and verify husbandry methodology available to conservationists and researchers.

## Methods

### Rearing

Foraging *B. vosnesenskii* gynes were net collected in low (27.43–76.2 m) and high (1,311.09–1,687.37 m) elevation sites in Oregon, USA, in 2022, deposited in 20-ml plastic collection vials with multiple 5-mm ventilation holes, and stored in an insulated container with ice packs (Table 1). A total of 117 gynes were collected from low-elevation sites in April 2022, and 58 gynes were collected from high-elevation sites in June 2022 (*n* = 175) (Table 1). After each collection event, the gynes were mailed in an insulated container with ice packs and access to cotton soaked with artificial nectar to the Ohio State University Department of Entomology Rothenbuhler Bee Research Lab in Columbus, OH, USA.

**Table 1.** Site information for *Bombus vosnesenskii* gynes collected in low- and high-elevation locations in Oregon, USA

Elevation (m)	Geographic coordinates	Gynes collected
Low elevation		**117**
27.43	45.426, −121.306	2
36.58	45.684, −121.401	31
51.82	45.685, −121.393	10
57.91	45.426, −123.305	12
57.91	45.711, −121.524	1
76.2	44.336, −123.172	61
High elevation		**58**
1,311.09	45.320, −121.622	11
1,466.09	44.225, −121.872	22
1,517.59	45.319, −121.653	3
1,530.71	43.931, −121.598	14
1,687.37	45.335, −121.663	8

**Fig. 1.**
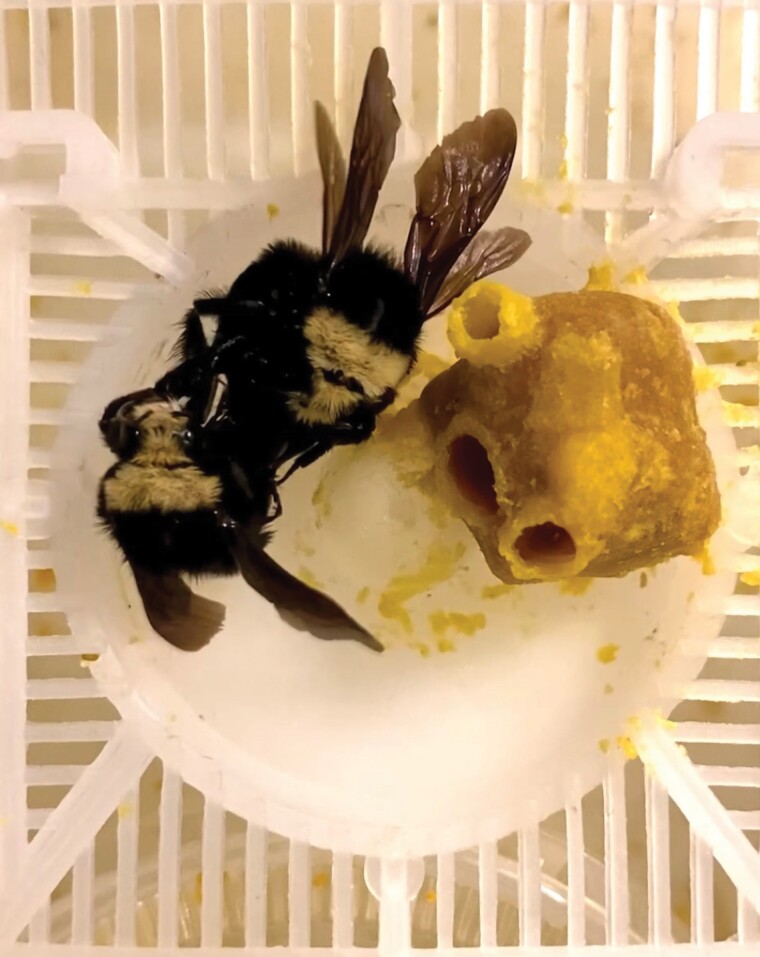
Open conflict among 2 *Bombus vosnesenskii* gynes during pleometrosis. During pleometrosis, the dominant gyne attacks and kills the other gyne, which increases oviposition success of the surviving gyne.

Following the methodology outlined in Rowe et al. (2023), the captured gynes were placed in plastic rearing units (15 × 15 × 10 cm; Biobest Canada, Leamington, ON, USA) in a designated rearing room maintained at 28 ± 2 °C and 65 ± 2% relative humidity in complete darkness. Red light was used when handling (i.e., feeding, cleaning, transferring) the colony to avoid light disturbance. At the lab, 26% of low-elevation gynes and 81% of high-elevation gynes were paired with conspecifics, which is known as co-founding or pleometrosis. Pleometrosis can serve as a source of social stress, often resulting in open conflict among the 2 gynes where one survives and the other perishes, increasing the oviposition and establishment success of the surviving gyne (Sladen 1912, Plowright and Jay 1966, Bernasconi and Keller 1996, Ptáček et al. 2000, Strange 2010) (Fig. 1). High-elevation gynes were paired with conspecifics more often to increase the likelihood of nest initiation, which was previously observed to be low for high-elevation gynes. Each gyne or pair of gynes was initially provided with a 2-g pollen provision (mixture of multi-floral honeybee collected pollen and artificial nectar) and a wicking feeder filled with artificial nectar (50% sugar solution with additives) (Rowe et al. 2023). Any gynes that failed to produce brood after 21 days were culled. After eclosion of the first worker, colonies were fed pollen and artificial nectar ad libitum. After eclosion of 5 workers, colonies were transferred to larger plastic rearing units (29 × 22 × 13 cm; Biobest Canada, Leamington, ON, Canada).

Colonies were assessed at least every 3 days over the course of their development to record days to first brood, days to first worker, days to 5 workers, days to 20 workers, and total emerged offspring (workers, males, and gynes). Nest initiation success (evidence of the queen to produce brood), nest establishment success (ability of the queen to rear one worker from brood), colony size (total emerged workers/males), and gyne production (total emerged gynes) of colonies produced from low- and high-elevation wild-caught *B. vosnesenskii* gynes was monitored in a controlled laboratory setting (Strange 2010, Strange et al. 2023). Additionally, average days to nest initiation and establishment were used to construct a timeline of colony development. Individual larval development within each colony was not assessed, so timelines are solely indicative of averaged colony development.

### Mating

**Fig. 2.**
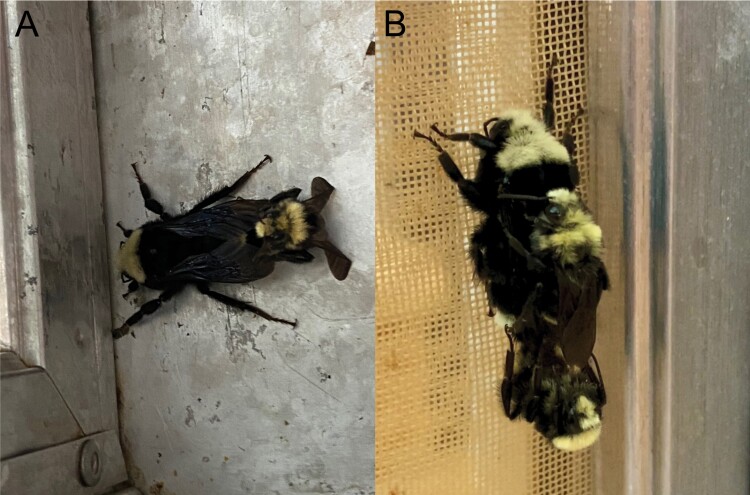
Coupling observed between *Bombus vosnesenskii* gynes and males: (A) gyne on left, male on right, and (B) gyne on the top, and males in the middle and bottom. Only the bottom male is mating with the gyne. The middle male is not actively involved in the mating process.

**Fig. 3.**
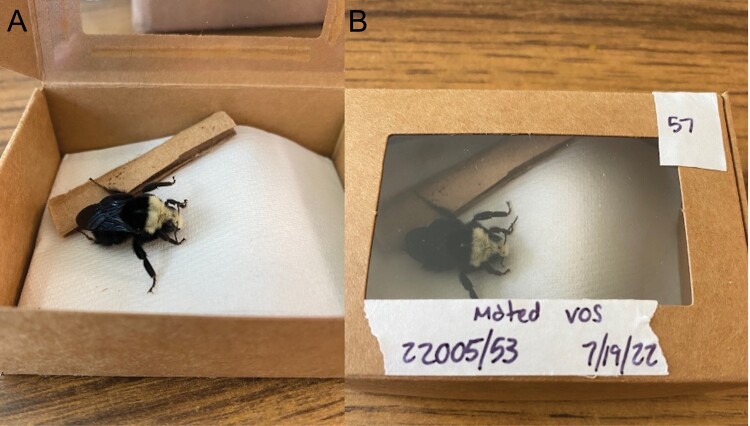
Overwintering set up for mated *Bombus vosnesenskii* gynes. A single gyne was placed into a mini paper craft box with a folded paper towel and a small strip of cardboard. Craft boxes with gynes were then placed into cold storage at either 1 or 6 °C to simulate overwintering.

To optimize mating success of *B. vosnesenskii* gynes produced from first-generation colonies, methodology followed Lindsay (2020) and Rowe et al. (2023). New gynes and males were extracted from each of the colonies every 48 h, kept in separate plastic rearing units, and fed pollen and artificial nectar *ad libitum* for 10 days to allow all individuals to reach sexual maturity (Tasei et al. 1998, Jung et al., 2001, Lee et al. 2002, Kwon et al. 2006, Amin et al. 2011, Herndon 2020, Rowe et al. 2023). Gynes and males from separate colonies were then paired at a 1:3 ratio (at a minimum) in benchtop insect-rearing cages that ranged from 0.03 to 0.23 m^3^ and supplied with 2-g pollen provisions and artificial nectar. Cages were monitored for mating activity, identified by the linking and unlinking of male and gyne genitalia (Fig. 2). Once copulation was observed, each pair was removed from the insect-rearing cage and placed into a 475-ml plastic container for continued observation. Start and end times for mating were recorded to obtain the duration of each mating event. Gynes not observed mating after 48 h were assumed to have mated outside of monitoring hours (20:00–08:00) and were thus retained for further analysis. Mated gynes were then placed in individual plastic rearing units in the rearing room and fed 2-g pollen provisions and artificial nectar for 3 days. After the 3-day period, the mated gynes were weighed and placed in mini paper craft boxes (8.5 × 6 × 3 cm) for overwintering in cold storage (Lindsay 2020; Fig. 3).

### Overwintering

Mated gynes were randomly selected for cold storage in either 1 or 6 °C in miniature refrigerators for 54 days to simulate overwintering (artificial hibernation) following Lindsay (2020) and Rowe et al. (2023). Gynes were checked weekly to assess overwintering survival rates, and any deceased gynes were removed and weighed. After 54 days, all surviving gynes were weighed and placed in individual plastic rearing units supplied with 2-g pollen provisions and artificial nectar within the rearing room to establish second-generation colonies. All second-generation colonies were reared from a single gyne (no co-founding colonies).

To further stimulate brood production, second-generation gynes were subjected to CO_2_ narcosis one week after removal from cold storage if brood production had not commenced. CO_2_ narcosis has been shown to bypass diapause, induce oogenesis, and initiate egg production, increasing bumble bee reproductive success and overall fitness in controlled laboratory settings (Röseler 1985, Tasei 1994, Amsalem et al. 2015, Amsalem and Grozinger 2017, Rowe et al. 2023). Gynes that had not yet produced brood were removed from their individual rearing units and temporarily transferred into empty rearing units, which were then placed inside a transparent plastic bag. The bag was then filled with CO_2_ and sealed. Once unconscious, the gynes were kept in this state for 30 min before being transferred back into their individual rearing units. This process was repeated 3 times over the course of a week on all gynes that had not yet initiated brood. After the third round of CO_2_ narcosis, the gynes were left alone for one week. This process was then repeated the following week on all gynes that had not produced brood. Gynes were then given 21 days after the sixth round of CO_2_ narcosis to produce brood. Any gynes that did not produce brood after this time were culled. Any queens that produced brood were reared following the same methodology described in the *Bombus vosnesenskii* Rearing section.

### Data Analysis

Statistical analyses were conducted in R version 4.3.1 (R Core Team 2022). Two-sample *z*-tests for proportions were conducted to test for differences in both nest initiation and establishment success between first-generation low- and high-elevation colonies. For both first-generation low- and high-elevation colonies, 2-sample *z*-tests for proportions were used to test for differences in both nest initiation and establishment success between colonies produced from a single queen or via co-founding. Individual analysis of variance (ANOVA) was conducted to test for differences in the total number of emerged workers/males and total number of emerged gynes among low- and high-elevation *B. vosnesenskii* colonies (*P* < 0.05). A *t*-test was used to evaluate differences in copulation duration between low- and high-elevation gynes. A 2-sample *z*-test for proportions was conducted to test for differences in survivorship between low- and high-elevation overwintered gynes. A *t*-test was used to evaluate the impact of the gynes starting mass (gram) on overwintering survival for low- and high-elevation gynes in 1 and 6 °C cold storage. For second-generation low-elevation colonies, a 2-sample *z*-test for proportions was used to examine differences in the influence of CO_2_ narcosis on nest initiation success. Additionally, 2-sample *z*-tests for proportions were conducted to test for differences in nest initiation and establishment success between gynes overwintered at 1 and 6 °C.

## Results

### Rearing: First Generation

Of the 125 wild-caught gynes installed in rearing units, 62.4% produced brood cells (nest initiation) and 43.2% had at least 1 worker eclose (nest establishment) (Table 2). High-elevation *B. vosnesenskii* gynes had significantly higher nest initiation (*z*-score = 1.92, df = 1, *P* = 0.02) and establishment success (*z*-score = 1.93, df = 1, *P* = 0.02) than low-elevation gynes.

**Table 2.** Rearing success of first-generation low- and high-elevation *Bombus vosnesenskii* colonies produced from a single queen or via co-founding. Nest initiation was defined as evidence of the queen to produce brood, while nest establishment was defined as the eclosion of a single worker. *Bombus vosnesenskii* colony development was defined as days to nest initiation ± SD, days to nest establishment ± SD, days to 5 workers ± SD, and days to 20 workers ± SD

Rearing technique	Nest initiation	Nest establishment	Days to first brood	Days to first worker	Days to 5 workers	Days to 20 workers
Low elevation						
Single	34/69 (49.3%)	21/69 (30.4%)	9.9 ± 8.3	43.5 ± 13.8	56.4 ± 12.7	71.0 ± 11.9
Co-founding	19/24 (79.2%)	14/24 (58.3%)	10.7 ± 12.1	37.6 ± 15.2	60.4 ± 20.8	67.7 ± 11.5
Combined	53/93 (56.9%)	35/93 (37.6%)	10.2 ± 9.7	41.2 ± 14.5	58.0 ± 16.2	69.9 ± 11.7
High elevation						
Single	4/6 (66.7%)	2/6 (33.3%)	8.0 ± 4.1	35.0 ± 7.1	44.0 ± 11.3	57.0 ± 11.3
Co-founding	21/26 (80.8%)	17/26 (65.4%)	8.2 ± 5.1	41.8 ± 15.2	57.4 ± 14.3	75.7 ± 14.1
Combined	25/32 (78.1%)	19/32 (59.4%)	8.2 ± 4.9	41.2 ± 14.6	55.8 ± 14.4	73.2 ± 14.9

For low-elevation wild-caught *B. vosnesenskii* gynes, 56.9% produced brood cells and 37.6% had at least one worker eclose (Table 2). Low-elevation gynes reared via co-founding had significantly higher nest initiation (*z*-score = 2.31, df = 1, *P* = 0.01) and nest establishment (*z*-score = 2.18, df = 1, *P* = 0.01) success compared to those reared from a single gyne. For low-elevation colonies reared from a single queen, 49.3% initiated nesting 9.9 ± 8.3 days after nest instalment, and 30.4% established nests 43.5 ± 13.8 days after nest instalment (Table 2). Meanwhile, 79.2% of low-elevation colonies reared via co-founding produced brood 10.7 ± 12.1 days after nest instalment, and 58.3% established nests 37.6 ± 15.2 days after nest instalment (Table 2).

We observed that 78.1% of high-elevation wild-caught *B. vosnesenskii* gynes produced brood and 59.4% had at least one worker eclose (Table 2). High-elevation gynes reared via co-founding tended to have higher nest initiation and establishment success per rearing unit compared to those reared from a single gyne. For high-elevation colonies reared from a single queen, 66.7% initiated nesting 8.0 ± 4.1 days after nest instalment, and 33.3% established nests 35.0 ± 7.1 days after nest instalment (Table 2). Meanwhile, 80.8% of high-selevation colonies reared via co-founding produced brood 8.2 ± 5.1 days after nest instalment, and 65.4% established nests within 41.8 ± 15.2 days after nest instalment (Table 2). Given differences in sample size among colonies reared from a single gyne or via co-founding, we could not statistically evaluate the impact of rearing technique on nest initiation and establishment success.

**Fig. 4.**
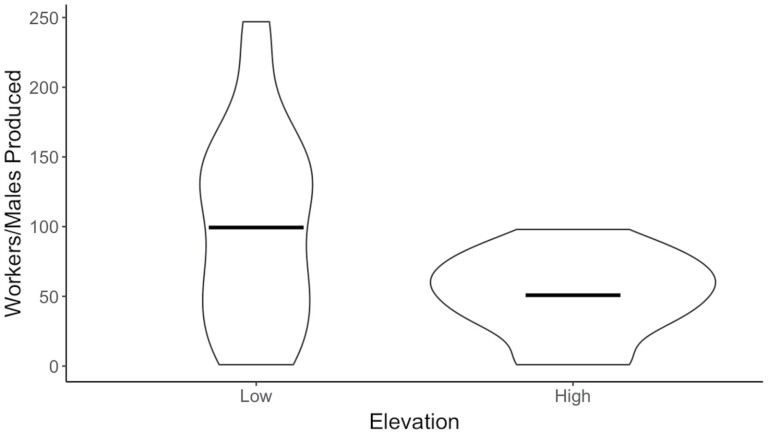
The total number of eclosed workers and males produced by low- and high-elevation *Bombus vosnesenskii* colonies, which is indicative of colony size. Low-elevation *B. vosnesenskii* queens produced larger colonies on average than high-elevation queens. Crossbars represent the mean.

**Fig. 5.**
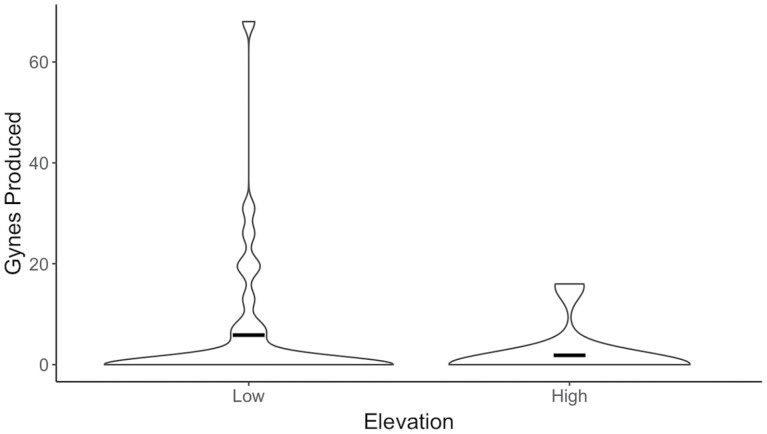
The total number of gynes produced by low- and high-elevation *Bombus vosnesenskii* colonies. The average number of gynes produced per colony did not differ between low- and high-elevation colonies. Crossbars represent the mean.

Overall, colony size and gyne production varied among low- and high-elevation *B. vosnesenskii* colonies. Low-elevation *B. vosnesenskii* had larger colonies with queens producing significantly more workers and males than those from high-elevation colonies (*F* = 8.48, df = 1, *P* = 0.005) (Fig. 4). Low-elevation queens produced an average of 99.4 ± 69.3 workers and males per colony, ranging from 1 to 247 offspring. High-elevation colonies ranged from 1 to 98 offspring, with an average production of 50.8 ± 29.2 workers and males. Gyne production did not differ significantly between low- and high-elevation colonies (*F* = 1.57, df = 1, *P* = 0.22) (Fig. 5). Low-elevation queens produced 5.9 ± 13.5 gynes on average with a maximum of 68 gynes produced from a single colony, whereas high-elevation queens produced an average of 1.8 ± 4.9 gynes with a maximum of 16 gynes produced from a single colony. Overall, 200 low-elevation gynes were produced by 11 colonies, while 37 high-elevation gynes were produced by 3 colonies.

### Mating

Copulation was recorded for 148 of the 200 low-elevation gynes. Mating duration for low-elevation gynes ranged from 3 min to 4 h and 19 min, with a mean duration of 1 h and 2 min. Additionally, copulation was recorded for 21 of the 37 high-elevation gynes. Mating duration for high-elevation gynes ranged from 3 min to 1 h and 36 min, with a mean duration of 51 min. Copulation duration between low- and high-elevation gynes was not significantly different (*t* = 1.75, df = 42.87, *P* = 0.09). The remaining 52 low-elevation and 16 high-elevation gynes not observed mating were not included in the copulation duration analysis but were evaluated for overwintering survival.

### Overwintering

Of the 231 mated gynes placed in cold storage at 1 or 6 °C, 65.4% survived overwintering after 54 days. High-elevation gynes had significantly higher survivorship than low-elevation gynes (*z*-score = 3.57, df = 1, *P* < 0.001), with 59.1% of low-elevation gynes and 91.9% high-elevation gynes surviving cold storage (Table 3).

**Table 3.** Overwintering survival of mated low- and high-elevation *B. vosnesenskii* gynes after 54 days in 1 or 6 °C cold storage

Elevation	1 °C cold storage survival rates	6 °C cold storage survival rates	Combined cold storage survival rates
Low	34/55 (61.8%)	57/99 (57.6%)	91/154 (59.1%)
High	18/19 (94.7%)	16/18 (88.9%)	34/37 (91.9%)

Mated low-elevation gynes (*n* = 194) were placed into cold storage to stimulate overwintering conditions: 95 at 1 °C and 99 at 6 °C. Due to a malfunction in the 1 °C refrigerator, temperatures reached −4 °C overnight causing 40 of the mated low-elevation gynes to die. These specimens were subsequently removed from data analysis. Nevertheless, 61.8% of mated low-elevation gynes survived overwintering in 1 °C cold storage and 57.6% survived overwintering in 6 °C cold storage (Table 3). Mated low-elevation gynes with higher body masses had significantly higher survival rates than those with lower masses in both 1 °C (*t* = 6.01, df = 35.99, *P* < 0.001) and 6 °C (*t* = 9.53, df = 60.75, *P* < 0.001) cold storage.

Mated high-elevation gynes (*n* = 37) were placed into cold storage to stimulate overwintering conditions: 19 at 1 °C and 18 at 6 °C. For mated high-elevation gynes, 94.7% and 88.9% survived overwintering in 1 and 6 °C cold storage, respectively (Table 3). Given the high survival rates of mated high-elevation gynes held at 1 °C, statistical comparisons could not be used to evaluate the impact of gyne starting mass on overwintering survival. For mated high-elevation gynes in 6 °C cold storage, survival rates were not influenced by mass (*t* = 1.26, df = 1.16, *P* = 0.41).

### Rearing: Second Generation

Of second-generation low-elevation *B. vosnesenskii* colonies reared from a single queen, 26.4% produced brood and 14.3% had at least one worker eclose (Table 4). Nesting success was not influenced by overwintering temperature as nest initiation (*z*-score = 0.23, df = 1, *P* = 0.41) and establishment (*z*-score = 0.08, df = 1, *P* = 0.5) success did not differ significantly between 1 and 6 °C. For low-elevation gynes overwintered at 1 °C, 23.5% initiated nesting 37.3 ± 14 days after nest installation and 14.7% established nests 64.2 ± 10.4 days after nest instalment (Table 4). Meanwhile, 28.1% of low-elevation gynes overwintered at 6 °C produced brood 48.3 ± 15.1 days after nest instalment and 14% established nests within 78.0 ± 9.8 days after nest instalment (Table 4). Furthermore, CO_2_ narcosis did not influence nest initiation success (*z*-score = 1.02, df = 1, *P* = 0.15). None of the 34 second-generation high-elevation gynes that survived overwintering conditions produced brood 36.4 ± 16.4 days after nest instalment and 6 rounds of CO_2_ narcosis.

**Table 4.** Rearing success of second-generation low-elevation *B. vosnesenskii* colonies produced from a single queen. Nest initiation was defined as evidence of the queen producing brood, while nest establishment was defined as the eclosion of a single worker. *Bombus vosnesenskii* colony development in captivity was defined as days to nest initiation ± SD, days to nest establishment ± SD, days to 5 workers ± SD, and days to 20 workers ± SD

Overwintering condition	Nest initiation	Nest establishment	Days to first brood	Days to first worker	Days to 5 workers	Days to 20 workers
Low elevation						
1 °C	8/34 (23.5%)	5/34 (14.7%)	37.3 ± 14.0	64.2 ± 10.4	72.3 ± 9.5	99.0 ± 0
6 °C	16/57 (28.1%)	8/57 (14.0%)	48.3 ± 15.1	78.0 ± 9.8	81.7 ± 5.7	92.0 ± 0
Combined	24/91 (26.4%)	13/91 (14.3%)	44.6 ± 15.4	72.7 ± 11.9	78.0 ± 8.2	95.5 ± 4.9

Overall, low-elevation queens produced an average of 9.2 ± 13.9 workers and males, ranging from 0 to 50 offspring, and 0.5 ± 1 gynes. A total of 6 gynes were produced by 3 second-generation colonies, with 3 gynes eclosing from a single colony.

## Discussion

Although *B. vosnesenskii* is commercially available in the United States, this is only the second study to publish information on rearing methods, nest success, developmental timelines, colony size, and gyne production for this species (Strange et al. 2023), and the first to document mating and overwintering success and methodology. Therefore, this comprehensive, observational study enhances our understanding of *B. vosnesenskii* nesting, mating, and overwintering biology from low and high elevations, which can be used by researchers and conservationists to address knowledge gaps and enhance captive rearing of bumble bees. Methods outlined in this study can also be employed when rearing other *Bombus* species, making this research broadly applicable to researchers and industry members.

First-generation *B. vosnesenskii* had high nest success in a controlled laboratory setting, which substantiates previous results obtained in a 5-yr study by Strange et al. (2023). Our study documented that 62.4% of *B. vosnesenskii* initiated nesting and 43.2% established nests, which was higher than the nest success recorded in Strange et al. (2023) (initiation: 48.2%; establishment: 25.2%). Although nest success was high across both elevations, high-elevation gynes had significantly higher nest initiation and establishment success than low-elevation gynes. Meanwhile, low-elevation colonies were significantly larger on average. Offspring production (low: 99.4 ± 69.3; high: 50.8 ± 29.2) in this study was much lower than the average 238.8 ± 151.6 workers/males documented in Strange et al. (2023). Furthermore, low- and high-elevation colonies produced a considerable number of gynes (low: *n* = 200, high: *n* = 37), indicating high production of sexually reproductive individuals. Future research should evaluate biological differences between low- and high-elevation *B. vosnesenskii* across a broad geographic range to elucidate factors that could impact the observed differences in nest establishment, colony size, and gyne production. For example, the difference in colony size from gynes collected from 2 elevations but reared in a common laboratory setting suggests that selection for colony size may instead be occurring across this elevational gradient due to genetic variation. Shorter seasons at high-elevation sites may be selected for the rapid development of colonies (Lozier et al. 2021), resulting in smaller colonies that are quicker to produce reproductive offspring to facilitate the continuation of the colony’s life cycle. Furthermore, as these results are only representative of a single year, future research should be conducted over multiple years to determine whether these findings are relatively consistent or whether annual variation occurs.

Documenting bumble bee mating in the wild is difficult due to the short time period in which coupling occurs and the fact that mating is a brief period within their life cycle. Therefore, assessing mating in captivity can provide information on variations in bumble bee mating behavior among species (Rowe et al. 2023). However, mating success is often low in captivity, despite advancements in methodology to maximize mating strategies (Tasei et al. 1998, Herndon 2020, Treanore et al. 2021, Rowe et al. 2023). Previous research documented that mating duration typically lasts 10–60 min (van Honk et al. 1978, Foster 1992, Duvoisin et al. 1999, Amin et al. 2009). Whereas in our study, mating ranged from 3 min to 4 h and 19 min across 169 *B. vosnesenskii* gynes. While the transfer of sperm to the spermatheca likely occurs within the first 2 min, the extended period in which the male remains coupled with the gyne is likely a behavioral adaptation to reduce multiple mating (Duvoisin et al. 1999, Baer et al. 2001, Sauter et al. 2001, Brown et al. 2002). While many bumble bee species appear to be monandrous based on molecular analyses (Estoup et al. 1995, Schmid-Hempel and Schmid-Hempel 2000), other bumble bee species are polyandrous, mating up to 6 times (Estoup et al. 1995, Schmid-Hempel and Schmid-Hempel 2000, Paxton et al. 2001). Regardless of whether the species is monandrous or polyandrous, males can impose monandry on gynes and reduce the probability of multiple mating by inserting a mating plug during copulation (Duvoisin et al. 1999, Baer et al. 2001, Sauter et al. 2001). It has been hypothesized that the longer the male remains attached, the probability that the plug will fully deposit and properly set within the gynes reproductive tract increases (Duvoisin et al. 1999, Brown et al. 2002). Previous research also suggests that as mating duration increases, the probability of remating decreases (Brown et al. 2002). While this may be due to the successful placement of the mating plug, it may also be due to the energy and time cost imposed on the gyne during mating, making her less likely to choose to remate (Brown et al. 2002). In the present study, *B. vosnesenskii* gynes were observed mating multiple times if the gyne was not removed from the benchtop rearing cage. However, whether the subsequent males were successful at transferring sperm to the spermatheca of the gyne is unknown. Furthermore, since mating was observed between 8:00 and 20:00 in this study, it is possible that multiple matings occurred outside of this time period, but were not documented. Future research is needed to evaluate the success of multiple mating activities and the efficacy of mating plugs in *B. vosnesenskii.* Determining the success of multiple mating activities would be accomplished by genotyping workers within the same colony to determine their paternity, which could represent the presence of one or multiple fathers.

Similar to mating success in captivity, cold storage is often associated with high mortality (Lindsay 2020), despite improvements in overwintering protocols (Rowe et al. 2023). Survival in cold storage has been found to decrease drastically after 3 months (Lindsay 2020), which is relatively short compared to the length of time mated gynes undergo overwintering in the wild (Rowe et al. 2023). Initial body mass has been found to impact overwintering survival, with heavier queens having an advantage due to an increase in the accumulation of lipids in the bumble bee’s fat body (Alford 1969, Holm 1972, Haunerland and Shirk 1995, Beekman et al. 1998, Fliszkiewicz and Wilkaniec 2007, Yoon et al. 2010, Lindsay 2020). We documented high success rates when overwintering gynes for 54 days, as 59.1% of low-elevation gynes and 91.9% of high-elevation gynes survived. Similar to previous findings, body mass significantly influenced mated low-elevation gyne overwintering survival, with heavier bees having higher survival rates in both 1 and 6 °C cold storage. However, body size did not significantly impact high-elevation gynes, as survival rates were high across both cold storage temperatures. This significant difference in survivorship across the 2 elevations may be a result of geographic differences and bioclimatic variables influencing their thermal abilities. Previous studies have identified that critical thermal minima decrease with increasing elevations, so high-elevation gynes may be more cold tolerant and less susceptible to cold shock than low-elevation gynes (Oyen et al. 2016, Gonzalez 2022), but see Oyen et al. (2021). Furthermore, our high-elevation gynes may have fared better in this study than low-elevation gynes since the thermal regime was set at a consistent temperature (Lindsay 2020). High-elevation *B. vosnesenskii* may be adapted to more consistent overwintering temperatures as increased snowpack can act as an insulator, reducing variation in soil temperatures (Lindsay 2020). Additionally, since snowpack remains for extended periods of time in high-elevation environments, high-elevation *B. vosnesenskii* may be adapted to survive longer in cold storage. This may have contributed to the high survival rates observed across both cold storage temperatures regardless of their initial body mass. While survival rates did not differ significantly when comparing cold storage temperatures, survivorship was higher in 1 °C cold storage than 6 °C for both low- and high-elevation gynes, which may reflect lower metabolic rates and therefore lower rates of energy usage at cooler temperatures (Heinrich 1974, Martinet et al. 2020).

Nest success was lower in the second-generation *B. vosnesenskii* than the first-generation *B. vosnesenskii*, with 26.4% of low-elevation gynes initiating nests and 14.3% establishing nests, and 0% of high-elevation gynes initiating nests. Second-generation nest success may improve with the implementation of co-founding, since first-generation gynes had higher nest initiation and establishment success per rearing unit compared to those reared from a single gyne. Additional research is needed to assess variables that may have contributed to the lack of nest initiation in high-elevation gynes, including time spent in cold storage, floral resource quality, and the successful transfer of sperm to the spermatheca. While the overwintered gynes were subjected to several rounds of CO_2_ narcosis to stimulate broodiness, these treatments did not improve nest initiation success. This differs from previous results, which found that CO_2_ narcosis induces oogenesis and initiates egg production, which can increase reproductive success and overall fitness in bumble bees (Röseler 1985, Tasei 1994, Amsalem et al. 2015, Amsalem and Grozinger 2017, Treanore et al. 2021, Rowe et al. 2023). Although nest success was lower, it is necessary to underscore the importance and accomplishment behind successfully rearing a second-generation of *B. vosnesenskii* colonies in a controlled laboratory setting.

With the rearing, mating, and overwintering methods employed in this study, we were able to achieve high first-generation nest success and gyne production, successful transfer of sperm to the spermatheca, and high overwintering survivorship. When considering the nest success obtained within both the first- and second-generation colonies, this is likely much higher within a controlled laboratory setting than what occurs in the wild. Bumble bees raised in captivity are protected from many external factors, such as variation in weather, exposure to pesticides, predation, reduced genetic variation, and restricted access to adequate floral and nesting resources, which can increase mortality and reduce overall health (Rowe et al. 2023). As such, captive rearing not only contributes to the enhanced knowledge of bumble bee biology but can also support bumble bee conservation by mitigating the effects of population declines via assisted reintroductions and increased genetic diversity.

## Supplementary Material

iead089_suppl_Supplementary_Tables

## Data Availability

The data and code supporting the findings of this study are openly available on Zenodo at https://doi.org/10.5281/zenodo.8161450.
