# Supplementary material for: It is buzziness time: rearing, mating, and overwintering Bombus vosnesenskii (Hymenoptera: Apidae)
Source: J Insect Sci. 2023 Oct 7;23(5):18. doi: 10.1093/jisesa/iead089 (PMC10560002; doi:10.1093/jisesa/iead089)
Supplement: iead089_suppl_Supplementary_Tables [file iead089_suppl_supplementary_tables.docx]

**Table 1.** Site information for *Bombus vosnesenskii* gynes collected in low and high elevation locations in Oregon, USA.

| **Elevation (m)** | **Geographic Coordinates** | **Gynes Collected** |
| --- | --- | --- |
| *Low Elevation* |  | **117** |
| 27.43 | 45.426, -121.306 | 2 |
| 36.58 | 45.684, -121.401 | 31 |
| 51.82 | 45.685, -121.393 | 10 |
| 57.91 | 45.426, -123.305 | 12 |
| 57.91 | 45.711, -121.524 | 1 |
| 76.2 | 44.336, -123.172 | 61 |
| *High Elevation* |  | **58** |
| 1311.09 | 45.320, -121.622 | 11 |
| 1466.09 | 44.225, -121.872 | 22 |
| 1517.59 | 45.319, -121.653 | 3 |
| 1530.71 | 43.931, -121.598 | 14 |
| 1687.37 | 45.335, -121.663 | 8 |

**Table 2.** Rearing success of first-generation low and high elevation *B.* *vosnesenskii* colonies produced from a single queen or via co-founding. Nest initiation was defined as evidence of the queen to produce brood, while nest establishment was defined as the eclosion of a single worker. *Bombus vosnesenskii* colony development was defined as days to nest initiation ± SD, days to nest establishment ± SD, days to five workers ± SD, and days to twenty workers ± SD.

| **Rearing**  **Technique** | **Nest**  **Initiation** | **Nest**  **Establishment** | **Days to**  **First**  **Brood** | **Days to**  **First**  **Worker** | **Days to**  **Five**  **Workers** | **Days to**  **Twenty**  **Workers** |
| --- | --- | --- | --- | --- | --- | --- |
| *Low Elevation* |  |  |  |  |  |  |
| Single | 34/69  (49.3%) | 21/69  (30.4%) | 9.9 ± 8.3 | 43.5 ± 13.8 | 56.4 ± 12.7 | 71.0 ± 11.9 |
| Co-founding | 19/24  (79.2%) | 14/24  (58.3%) | 10.7 ± 12.1 | 37.6 ± 15.2 | 60.4 ± 20.8 | 67.7 ± 11.5 |
| Combined | 53/93  (56.9%) | 35/93  (37.6%) | 10.2 ± 9.7 | 41.2 ± 14.5 | 58.0 ± 16.2 | 69.9 ± 11.7 |
| *High Elevation* |  |  |  |  |  |  |
| Single | 4/6  (66.7%) | 2/6  (33.3%) | 8.0 ± 4.1 | 35.0 ± 7.1 | 44.0 ± 11.3 | 57.0 ± 11.3 |
| Co-founding | 21/26  (80.8%) | 17/26  (65.4%) | 8.2 ± 5.1 | 41.8 ± 15.2 | 57.4 ± 14.3 | 75.7 ± 14.1 |
| Combined | 25/32  (78.1%) | 19/32  (59.4%) | 8.2 ± 4.9 | 41.2 ± 14.6 | 55.8 ± 14.4 | 73.2 ± 14.9 |

**Table 3.** Overwintering survival of mated low and high elevation *B.* *vosnesenskii* gynes after 54 days in 1ºC or 6ºC cold storage.

| **Elevation** | **1ºC Cold Storage Survival Rates** | **6ºC Cold Storage Survival Rates** | **Combined Cold Storage Survival Rates** |
| --- | --- | --- | --- |
| Low | 34/55  (61.8%) | 57/99  (57.6%) | 91/154  (59.1%) |
| High | 18/19  (94.7%) | 16/18  (88.9%) | 34/37  (91.9%) |

**Table 4.** Rearing success of second-generation low elevation *B.* *vosnesenskii* colonies produced from a single queen. Nest initiation was defined as evidence of the queen to produce brood, while nest establishment was defined as the eclosion of a single worker. *Bombus vosnesenskii* colony development in captivity was defined as days to nest initiation ± SD, days to nest establishment ± SD, days to five workers ± SD, and days to twenty workers ± SD.

| **Overwintering**  **Condition** | **Nest**  **Initiation** | **Nest**  **Establishment** | **Days to**  **First**  **Brood** | **Days to**  **First**  **Worker** | **Days to**  **Five**  **Workers** | **Days to**  **Twenty**  **Workers** |
| --- | --- | --- | --- | --- | --- | --- |
| *Low Elevation* |  |  |  |  |  |  |
| 1ºC | 8/34  (23.5%) | 5/34  (14.7%) | 37.3 ± 14.0 | 64.2 ± 10.4 | 72.3 ± 9.5 | 99.0 ± 0 |
| 6ºC | 16/57  (28.1%) | 8/57  (14.0%) | 48.3 ± 15.1 | 78.0 ± 9.8 | 81.7 ± 5.7 | 92.0 ± 0 |
| Combined | 24/91  (26.4%) | 13/91  (14.3%) | 44.6 ± 15.4 | 72.7 ± 11.9 | 78.0 ± 8.2 | 95.5 ± 4.9 |
